# Frequency of anaplastic lymphoma kinase rearrangements in Moroccan patients with non small cell lung cancer: a multi-institutional national retrospective study

**DOI:** 10.1186/s12885-020-06973-4

**Published:** 2020-05-27

**Authors:** Hind El yacoubi, Mohamed Lemine Sow, Fouad Kettani, Lamia Gamra, Amina Mestari, Lamia Jabri, Ibrahim Elghissassi, Hassan Errihani

**Affiliations:** 1 Research and investigation in medical oncology Moroccan group, Faculty of medicine and pharmacy, Mohammed V University, Impasse souissi, 10100 Rabat, Morocco; 2 Department of Medical Oncology, Mohammed Bouafi Hospital, Casablanca, Morocco; 3 Department of Medical Oncology, National Institute of Oncology, Rabat, Morocco; 4 Department of Pathology, Nations-Unies Pathology Center, Rabat, Morocco; 5 Department of Pathology, Hassan Pathology Center, Rabat, Morocco; 6 Department of Pathology, Agdal Pathology Center, Rabat, Morocco; 7 Department of Pathology, Casapath Pathology Center, Casablanca, Morocco

**Keywords:** Anaplastic lymphoma kinase rearrangement, Non small cell lung cancer, Morroco, Frequency

## Abstract

**Background:**

Anaplastic lymphoma kinase (ALK) rearrangement is a predictive factor of response to ALK inhibitors in non small cell lung cancer (NSCLC). The prevalence of ALK rearrangements is well known in Whites and Asians. However, data identifying the frequency of this rearrangement in Moroccan and North African population are lacking. The objective of this study is to report the frequency of ALK rearrangement in a group of Moroccan patients with NSCLC.

**Methods:**

A retrospective study was performed enrolling 120 Moroccan patients with NSCLC whose biopsy samples were tested for ALK rearrangement in order to identify the frequency of ALK rearrangement and its potential association with selected variables. The ALK testing was established using fluorescent in situ hybridization (FISH) or immunohistochemistry (IHC).

**Results:**

The frequency of ALK rearrangement was 4.2% (5/120). All positive cases were males with advanced adenocarcinoma. ALK rearrangements prevalence was significantly higher in older patients.

**Conclusions:**

The frequency of ALK rearrangements among the Moroccan population tends to correlate with the average frequency reported worldwide, with some specific features. Further prospective studies with larger patients’ numbers are needed to verify these findings.

## Background

Lung cancer is one of the most commonly diagnosed cancers in both sexes and the leading cause of cancer deaths worldwide [1]. Nevertheless, several advances have improved the management and treatment of non small cell lung cancer (NSCLC), mainly the discovery of specific oncogenic drivers that guide the choice of treatment towards the appropriate targeted therapy. Anaplastic lymphoma kinase (ALK) rearrangement was first documented in 2007 in NSCLC [2]. Echinoderm Microtubule associated protein Like 4 (EML4) gene is the most frequent ALK partner that have been described among various ALK fusion genes [3]. ALK-EML4 rearrangement is the consequence of a gene fusion in chromosome 2 between ALK gene and EML4 gene which leads to the activation of the tyrosine kinase domain of ALK protein that enhances tumor growth and proliferation [2]. Thus, targeting this tumorigenesis pathway has improved the survival and clinical outcomes of this subset of patients harboring ALK rearrangements by using ALK inhibitors [4, 5]. However, ALK rearrangements occur in 2 to 7% of all NSCLC and this frequency may vary by race, gender, histology type, and smoking habits [6–8]. These literature data are based on studies in Whites and Asians, the epidemiology of ALK rearrangements remains, nevertheless, less known in North African population.

The aim of this study is to report the frequency of ALK rearrangements in a group of Moroccan patients with NSCLC. Moreover, the study investigates the potential associations between ALK rearrangement status and selected variables.

## Methods

A retrospective study was performed enrolling all NSCLC cases whose biopsy samples were tested for ALK rearrangement status between January 2014 and December 2017, from four pathology centers in Morocco.

The histopathology diagnosis was confirmed in local laboratories, then the formalin-fixed paraffin embedded tumor tissues were sent to France to test the ALK rearrangement using fluorescent in situ hybridization (FISH) or immunohistochemistry (IHC). The FISH analyses were performed in Biomnis medical biology laboratory in Lyon using LSI ALK 2p23 Dual Color, Break Apart Rearrangement Probe (Abott-Vysis). One hundred nuclei have to be counted to detect ALK rearrangements. The Tumor tissues were considered ALK FISH-positive if more than 15% of tumor cells are ALK rearranged. Otherwise the samples were considered as ALK FISH-negative. The IHC analyses were performed in the genetic laboratory of tumors at the Rouen university hospital using the antibody D5F3 clon (Roche-Ventana). IHC staining scores were assessed as follows: 0, (no staining); 1+ (faint cytoplasmic staining); 2+ (moderate cytoplasmic staining); and 3+ (intense granular cytoplasmic staining). IHC score 0 defined negative ALK rearrangements. Tumor tissues scored 3+ were considered as positive ALK rearrangements. IHC scores 1+ and 2+ were considered as equivocal cases. The confirmation of ALK status was based on FISH results or IHC scores 0 and 3, only equivocal cases had to be re-evaluate by FISH, as per institutional and national policy and guidelines for ALK testing.

Genetic analysis of epidermal growth factor receptor (EGFR) and Kirsten rat sarcoma (Kras) gene mutations was performed using polymerase chain reaction assay and direct sequencing of exons 18, 19, 20, and 21of the EGFR gene and exon 2 of Kras gene.

The study included only Moroccan patients with advanced NSCLC. All ALK tests obtained from the participating pathology labs were included. Information about age, gender, smoking history, histopathology type, ALK rearrangement status, EGFR and Kras mutations were collected. Patients who had smoked < 100 cigarettes in their lifetime were defined as non-smokers and all others as smokers.

The data were described using standard descriptive statistical methods. Fisher’s exact test was used to analyze the potential combinations of ALK rearrangement with gender, smoking status, histopathology type, the ALK test method performed, and EGFR and Kras status. The *t* test was used to compare age means. A *p*-value less than 0.05 was considered statistically significant. The *Statistical Package for the Social Sciences* (SPSS) (version 20; SPSS Inc., Chicago, IL) was used to perform all the analyses.

## Results

One hundred twenty Moroccan patients with confirmed NSCLC were included. All tumor specimens were tested for ALK rearrangement. Ninety-two patients were males (76.7%), and twenty-eight were females (23.3%). The median age was 63 (range, 28–88) years. 87.5% presented with adenocarcinoma histology and 12.5% a mixed histology showing a partial adenocarcinoma differentiation, thus eligible for ALK testing. The ALK rearrangement test was performed mostly using FISH method (75%) as it is the gold standard of ALK testing. 90% of IHC tests carried out were conclusive (24 score 0, 3 score 3+). Three cases were equivocal and ALK FISH-negative. The baseline characteristics of the study population are summarized in Table 1.

**Table 1:** Demographic Characteristics of the study population

Characteristics	All patients (*n* = 120) N (%)
Age (yr)	
Median	63
Range	28–88
Gender	
Male	92 (76.7%)
Female	28 (23.3%)
Smoking status	
Smoker	68 (56.7%)
Non-smoker	52 (43.3%)
Histology	
Adenocarcinoma	105 (87.5%)
Others NSCLC	15 (12.5%)
Stage	
IV	120 (100%)
ALK test	
FISH	90 (75%)
IHC	30 (25%)
EGFR status	
Mutated	21 (17.5%)
Wild	99 (82.5%)
Kras status	
Mutated	7 (5.8%)
Wild	55 (45.8%)
Not tested	58 (48.3%)

*Yr* year, *NSCLC* non small cell lung cancer, *ALK* anaplastic lymphoma kinase, *FISH* fluorescent in situ hybridization, *IHC* immunohistochemistry, *EGFR* epidermal growth factor receptor, *Kras* Kirsten rat sarcoma

ALK rearrangement was found in 5 cases (4.2%) of the 120 patients included. All ALK positive patients were males with adenocarcinoma histology. Three were non-smokers and two were smokers. The ALK rearrangement was established using FISH in two cases and IHC in three cases (score 3+) (Table 2).

**Table 2:** Data of Patients with ALK Positive Rearrangement

Patients	Age-ranges	Gender	Smoking Status	Histology	ALK test
1	66–70	M	Smoker	Adenocarcinoma	FISH
2		M	Non-smoker	Adenocarcinoma	FISH
3		M	Smoker	Adenocarcinoma	IHC
4		M	Non-smoker	Adenocarcinoma	IHC
5		M	Non-smoker	Adenocarcinoma	IHC

*ALK* anaplastic lymphoma kinase, *M* male, *FISH* fluorescent in situ hybridization, *IHC* immunohistochemistry

The mean age of patients harboring positive ALK rearrangements was found to be higher than that of patients having negative ALK rearrangement, a difference which was statistically significant (67.8 versus 63.3 *p*<0.0001). There was no difference in the incidence of ALK rearrangement between males and females, smokers and non-smokers. No concomitant ALK, EGFR or Kras mutations were found. EGFR and Kras status did not influence the incidence of ALK rearrangement (Table 3).

**Table 3:** Patient Characteristics by ALK Rearrangement Status

Characteristics	ALK Rearrangement Status N(%)		P
	Negative	Positive	
Total	115 (95.8%)	5 (4.2%)	
Mean age at diagnosis (yr)	63.3	67.8	<0.0001*
Gender			0.58**
Male	87 (75.7%)	5 (100%)	
Female	28 (24.3%)	0 (0%)	
Smoking history			0.65**
Smoker	66 (57.4%)	2 (40%)	
Non-smoker	49 (42.6%)	3 (60%)	
Histological type			0.50**
Adenocarcinoma	100 (87%)	5 (100%)	
Others	15 (13%)	0 (0%)	
ALK test			0.09**
FISH	88 (76.5%)	2 (40%)	
IHC	27 (23.5%)	3 (60%)	
EGFR status			0.58**
Mutated	21 (100%)	0 (0%)	
Wild	94 (94.9%)	5 (5.1%)	
Kras status			0.69**
Mutated	7 (100%)	0 (0%)	
Wild	52 (94.5%)	3 (5.5%)	

*p value calculated by *t* test

**p value calculated by Fisher’s exact test

*ALK* anaplastic lymphoma kinase, *FISH* fluorescent in situ hybridization, *IHC* immunohistochemistry, *EGFR* epidermal growth factor receptor, *Kras* Kirsten rat sarcoma

## Discussion

Lung cancer is one of the most diagnosed cancers and the first cause of mortality related to cancer in both sexes [1]. Although important progress has been made in NSCLC molecular typing and personalized targeted therapies which has contributed to better clinical outcomes. The use of ALK inhibitors in patients with NSCLC harboring ALK rearrangement demonstrated impressive response rate and progression free survival compared to chemotherapy [4, 5]. Indeed the determination of ALK rearrangement status is a decisive factor of treatment and prognosis in NSCLC.

In our series ALK rearrangement was present in 5 of 120 Moroccan patients (4.2%). The present study is – to our knowledge- the first to report the ALK rearrangement frequency in Moroccan population and the largest in North Africa.

The frequency that we found falls globally between the frequencies reported in whites and those reported in Asians [9–14]. However, it’s lower than the frequency found in two previous Tunisian studies that reported an ALK rearrangement frequency of 5.2 and 9.09%, respectively [15, 16] (Table 4).

**Table 4:** Frequency of ALK rearrangement by race

Study	Race	ALK rearrangement (%)
Zhou et al. [9]	East Asians (China)	28/488 (5.7%)
Soda et al. [10]	East Asians (Japan)	32/754 (4.24%)
Jin et al. [11]	East Asians (Korea)	10/167 (6%)
Martinez et al. [12]	Whites (Spain)	7/99 (7.1%)
Yamaguchi et al. [13]	Whites (USA)	15/192 (7.81)
Desai et al. [14]	South Asians (India)	5/187 (2.7%)
Arfaoui et al. [15]	North Africans (Tunisia)	1/19 (5.2%)
Toumi et al. [16]	North Africans (Tunisia)	2/22 (9.09%)
This study	North Africans (Morocco)	5/120 (4.2%)

*ALK* anaplastic lymphoma kinase

Furthermore, previous studies found similar overall distributions of ALK rearrangements according to race, and showed that ALK rearrangement onset on NSCLC patients was less influenced by ethnicity [13, 14].

Fan et al. [6] reported in their meta-analysis that patients with ALK rearrangements were younger than those without, in both Asians and Whites. In our series we observed that Moroccan patients with ALK positive were older than those with ALK negative, the mean age was 67.8 and 63.3 years old respectively (*p*<0.0001). However, our findings are based on limited number of patients as compared to large published studies so we are unable to conclude in a real discordance with most available literature about age and ALK status [2, 6, 7].

On the whole, data in the literature about the relationship between sex and NSCLC ALK status are heterogeneous. Zhou et al. [9] reported a higher rate of ALK positive in female in an East Asian population, while Shaw et al. [7] found more ALK positive in males than females. Besides, the meta-analysis by Fan et al. [6] didn’t demonstrate a significant difference in ALK occurrence according to sex in a Western population, whereas Asian population presented more ALK positive in females than males. In the present study all ALK positive patients were males, without significant statistical difference according to patient’s sex, which is likely due to the fact that males outnumber females in our study group (76.7% versus 23.3%, respectively).

Several studies have shown that NSCLC patients harboring ALK rearrangement were more likely to be never smokers compared to negative ALK patients [6, 7, 9]. Chapman et al. [8] also confirmed in their meta-analysis that ALK rearrangements occurred more in never smokers than ever smoker in both Asian and White patients with NSCLC. Our present study reported more non-smokers in ALK positive patients (60%) but did not find a significant influence of smoking status on ALK positive frequency in Moroccan NSCLC patients, a result which is inconsistent with the majority of reports in the literature [6–9].

Histologically, previous studies reported more ALK fusions in patients with lung adenocarcinoma [5, 7, 8]. Fan et al. [6] found an association race dependent between lung cancer histology and ALK rearrangements. They identified a significantly higher proportion of ALK fusions in lung adenocarcinoma in Asians compared to Whites. In our group of Moroccan patients, among 120 included cases, 105 presented an adenocarcinoma. There was no relationship between the histological type and ALK status, a finding that tends to meet literature reports in White populations [6].

No coexistence of ALK rearrangement and EGFR or Kras mutations was detected in our series, a finding which is similar to widely reported mutually exclusive mutational pattern [17, 18]. However, recent large studies report a higher frequency of the concomitance of ALK rearrangement in NSCLC patients, especially whose harboring EGFR mutations [19, 20].

Even though the ALK rearrangement could change according to patient’s clinicopathological features, it is obvious that only positive ALK rearrangement is the predictive factor of clinical response to ALK inhibitors [4, 5]. Our findings should be interpreted with caution since we were limited by the small number of patients, the retrospective enrollment and the lack of data about received treatments. Although, little data exists on the mapping of ALK rearrangements for NSCLC in North Africa, the present study helps to characterize this subset of NSCLC in our region and hopefully will be followed by prospective studies.

## Conclusions

In summary, the present study found that the frequency of ALK rearrangement in Moroccan population with NSCLC goes along with the average frequency reported in unselected populations with NSCLC. In addition, older age might be a specific feature of Moroccan patients with ALK rearrangement. Further prospective studies with larger patient’s numbers are needed to verify these findings.

## Data Availability

The datasets used and/or analyzed during the current study are available from the corresponding author on reasonable request.

## Abbreviations

ALK: Anaplastic lymphoma kinase
EML4: Echinoderm Microtubule associated protein Like 4
EGFR: Epidermal growth factor receptor
FISH: Fluorescent in situ hybridization
IHC: Immunohistochemistry
Kras: Kirsten rat sarcoma
NSCLC: Non small cell lung cancer
